# Targeting NLRP3 Inflammasome Alleviates Synovitis by Reducing Pyroptosis in Rats with Experimental Temporomandibular Joint Osteoarthritis

**DOI:** 10.1155/2022/2581151

**Published:** 2022-11-23

**Authors:** Yinzi Xin, Wei Wang, Enyu Mao, Hefeng Yang, Song Li

**Affiliations:** ^1^Department of Orthodontics, Kunming Medical University School and Hospital of Stomatology, Kunming 650106, China; ^2^Yunnan Key Laboratory of Stomatology, Kunming 650106, China

## Abstract

The mechanism of temporomandibular joint osteoarthritis (TMJOA), which leads to the final erosion of cartilage and subchondral bone, has been widely demonstrated, but still not clearly elucidated. Many studies have pointed that NLRP3-mediated inflammation played a vital role in degenerative diseases. However, its interaction with synovitis of TMJOA has remained poorly investigated. In our study, we explored the role of NLRP3 inflammasome in TMJOA synovitis and the therapeutic potential of caspase-1 and NLRP3 inhibitors. By establishing a rat TMJOA model, we found that NLRP3 was upregulated in synovial tissue of TMJOA. It was involved in the progress of a programmed cell death called pyroptosis, which was caspase-1 dependent and ultimately triggered inflammatory mediator interleukin IL-1*β* release. Treatment with Ac-YVAD-cmk and MCC950, inhibitors targeting caspase-1 and NLRP3, respectively, significantly suppressed pyroptosis in TMJOA synovial tissue. Then, a macrophage- and fibroblast-like synoviocyte (FLS) cocultured model further verified the above results. Macrophage somehow promoted FLS pyroptosis in this study. Our results suggested that the NLRP3 inflammasome-mediated pyroptosis participated in synovial inflammation of TMJOA. Interfering with the progress could be a potential option for controlling TMJOA development.

## 1. Introduction

Temporomandibular joint osteoarthritis (TMJOA) is a degenerative disorder accompanied by symptoms of chronic pain and dysfunction of joint. TMJOA is one of the most severe type of temporomandibular disorders (TMD) affecting all age groups, especially prevalent in female patients. Progressive synovitis, cartilage degeneration, and subchondral bone remodeling are major pathological changes in most TMJOA cases [1]. Although the mechanism underlying the development of TMJOA remains unclear, substantial evidence revealed that TMJ synovial inflammation might be an important change initiating the OA progression and relevant with pain, joint dysfunction, and rapid cartilage erosion [2–4].

Temporomandibular synoviocytes including fibroblast-like synoviocyte (FLS) and macrophage-like synoviocyte (MLS) are major cellular components constituting the synovial lining [2, 4]. During OA progression, synoviocytes can secrete various proinflammatory cytokines and chemokines into the synovial fluid [2, 5]. These cytokines, such as tumor necrosis factor- (TNF-) alpha (TNF-*α*) and interleukin-1beta (IL-1*β*), contribute to local synovial inflammation and articular matrix degradation, which in turn expand inflammation by mediating more inflammation factor release [3].

Maturation and secretion of IL-1*β* require cleavage of pro-IL-1*β*, which is regulated by inflammatory caspases. Active caspases form within molecular platforms called inflammasomes. Inflammasomes play important role in many diseases, such as cardiovascular diseases, neurological disorders, oral disease, and inflammatory and autoimmune diseases [6–11]. The NLRP3 inflammasome is one of the most widely researched canonical inflammasomes. It consists of an inflammasome sensor protein, a central adaptor ASC (apoptosis-associated speck-like protein containing a caspase recruitment domain (CARD)), and pro-caspase-1. NLRP3 forms following recognition of two patterns PAMPs or DAMPs. The oligomerization of NLRP3 receptor and ASC in the cytosol activates recruiting process. The assembled NLRP3 inflammasome then structurally changes pro-caspase-1 into caspase-1 and ultimately stimulates active gasdermin D (GSDMD). Cleavage of GSDMD can lead to pyroptosis, a form of programmed cell death. Pyroptosis may aggravate inflammatory response by forming transmembrane pores and plasma membranes rupture, resulting in release of IL-1*β*, IL-18, and cascaded cytokines [12–14].

The function of NLRP3 inflammasome has been reported in the pathogenesis of many arthritic diseases, such as gout, knee osteoarthritis, and rheumatic arthritis [15–17]. Due to the unique anatomy of TMJ, whether NLRP3 inflammasome primes temporomandibular synoviocytes and mediates pyroptosis and subsequential inflammation in the TMJOA remains unclear.

In this study, we use a rat TMJOA model to explore whether NLRP3 inflammasome-mediated pyroptosis is involved in the process of TMJOA, especially the initial synovial inflammation. By using MCC950 and Ac-YVAD-cmk, two highly selective inhibitors targeting NLRP3 and caspase-1, respectively, we aim to demonstrate the therapeutic effect of alleviating pyroptosis on TMJ synovial inflammation reaction. These results may help us have a better understanding of the mechanism underlying TMJOA occurrence and provide more perspectives regarding TMJOA prevention and treatment.

## 2. Materials and Methods

### 2.1. Animals

Sprague-Dawley rats weighted 300–350 g were purchased from the Experimental Animal Center of Kunming Medical University. 60 male adult rats were kept under controlled temperature on a 12 h light/dark cycle in the specific pathogen-free room. Rats had free access to water and food. Animal experiments in the study were approved by the Animal Care and Use Committee of the Kunming Medical University (approval No. KMMU2020028). Experimental protocols were executed following the National Institute of Health Guide for the care and use of laboratory animals.

### 2.2. CFA Administration and Induction of TMJ Inflammation

For different experimental purposes, group assignments were randomly made. The rats were intraperitoneally anesthetized with sodium pentobarbital (50 mg/kg body weight). As a novel approach, TMJ intracapsular inflammation was induced by bilateral injection with 50 *μ*l complete Freund's adjuvant (CFA) (Sigma-Aldrich, St. Louis, MO, USA) (oil/saline at ratio of 1 : 1) [18–20]. 50 *μ*l saline was administered in the control group. Physical examination and histological analysis were used for evaluation of TMJ inflammation.

### 2.3. Application of Inhibitors

**Figure 1:**
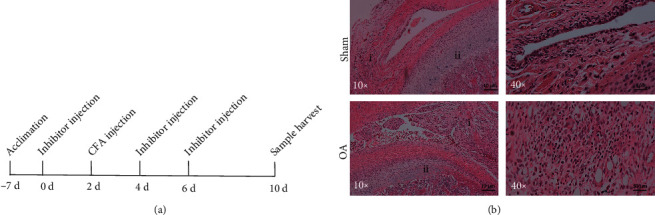
Time course and inflammation changes after intra-articular injection of CFA in rats. (a) Time course and procedures of CFA and inhibitor administration. (b) Representative changes of the synovium and condylar cartilage in the control and inflammatory tissues under H&E staining. (i) Synovium: proliferated synovial lining featured villous hyperplasia into cavity in OA group. (ii) Compared to the sham-treated group, mandibular condyle: proliferative layer, maturation layer, and hypertrophic layer were thinned in the OA group.

The caspase-1 inhibitor Ac-YVAD-cmk (Sigma-Aldrich, St. Louis, MO, USA) was dissolved in DMSO and diluted to 100 ng/*μ*l with saline. The rats in the AYC group were intra-articularly injected with 50 *μ*l caspase-1 inhibitor in both sides of TMJ. Equal volume of saline and DMSO was given in the OA group. The NLRP3 inhibitor MCC950 (MedChemExpress, Monmouth, NJ, USA) was diluted to 2 mg/ml with sterile saline and injected 10 mg/kg intraperitoneally. The OA group was treated with the same dose of sterilized saline. Drugs were injected initially 2 days prior CFA injection and repeated every 2 days for a week (Figure 1(a)).

### 2.4. Cell Cultures

Rat synovial tissues of TMJ were minced into 1~2 mm^2^ pieces and kept in 0.25% trypsin. Minced synovial tissues were then transferred into T-25 culture flask with DMEM supplemented with 10% foetal bovine serum (Gibco; Thermo Fisher Scientific, Waltham, MA, USA), 2 mM glutamine, and antibiotics (100 U/ml penicillin and 100 *μ*g/ml streptomycin) (Invitrogen, Carlsbad, CA, USA) for 1 week. When fibroblasts grew out from the tissue, the cells were assessed by morphology and immunofluorescence. Experiments were implemented using 3-6 passages of cultured synovial cells.

The THP-1 cells were maintained in RPMI-1640 medium (Hyclone, South Logan, Utah, USA). The cells were passaged every 2–3 days to maintain proper cell density of 5 × 10^5^ cells/ml. 50 ng/ml phorbol myristate acetate (PMA) (Sigma, St Louis, MO, USA) was used to differentiate cells into M0 macrophages in 48 h.

### 2.5. Coculture of Macrophages and FLS Cells

Transwell 6-well plate (3452; Corning, NY, USA) was used for coculture cells. FLS cells with density of 1 × 10^5^ cells/ml were cultured on the lower wells. The THP-1 cells were differentiated with PMA first and detached by Accutase (A6964; Sigma-Aldrich, Merck, Germany). Then, macrophages were resuspended in serum-free DMEM and added to the upper wells at a concentration of 1 × 10^5^ cells/ml. The cells were cocultured in a humidified atmosphere of 5% CO_2_ at 37°C for at least 24 h.

### 2.6. LPS+ATP Induction

Prior to stimulation, starve cells with serum-free DMEM for 24 h. Then, 1 *μ*g/ml lipopolysaccharide (LPS) was used to stimulate the inflammatory reactions for 12 h. 20 *μ*M Ac-YVAD-cmk or 10 *μ*M MCC950 was simultaneously added to the cells for inhibitory purpose. For the last 2 h, the cells were challenged with 3 mM adenosine triphosphate (ATP). Saline was served as controls. Supernatants and cell lysates were then collected for the following experiments.

### 2.7. Histological Analysis

TMJ tissues were harvested, fixed in 4% paraformaldehyde for 24 h, decalcified with EDTA (pH 7.4) for 2 weeks, and dehydrated and embedded in paraffin. Sliced into 4 *μ*m thick, TMJ paraffin sections were prepared for hematoxylin and eosin (H&E) staining.

For immunohistochemical analysis, sections were deparaffinized and dehydrated with decreasing concentration ethanol. Repair antigens using 0.01 M citrate buffer at 95°C and cool down to room temperature. Slices were blocked with 3% H_2_O_2_ for 5 min to make endogenous peroxidase inactive and incubated overnight with primary antibodies including NLRP3 (1 : 100, ET1610-93, Huabio), ASC (1 : 100, DF6304, Affinity), GSDMD (1 : 100, AF4012), caspase-1 (1 : 100, ET1608-69, Huabio), Affinity), and IL-1*β* (1 : 100, AF5103, Affinity) at 4°C. Then, the sections were washed with PBS and incubated with HRP-conjugated secondary antibodies for 15 min and DAB for 5 min. The immunoreaction was observed by a Zeiss microscope (Carl Zeiss MicroImaging) and quantified by using ImageJ software (NIH).

### 2.8. Terminal Deoxynucleotidyl Transferase dUTP Nick End Labeling (TUNEL) Immunofluorescence Assay

After deparaffinizing tissue section, the TUNEL immunofluorescence detection kit (Roche Holding AG, Basel Switzerland) was utilized to label DNA strand breaks according to the manual. Then sections were washed in PBS and double stained with DAPI. Images of TMJ slices were taken under a fluorescence microscope (Nikon, Tokyo, Japan).

### 2.9. Immunofluorescence

The cells were fixed in 4% paraformaldehyde and permeabilised in 0.5% Triton X-100 for 15 min, respectively, at room temperature. Incubate the cells with diluted primary antibody against rat vimentin (1: 200, Ab92547, Abcam) overnight at 4°C. Diluted fluorescent secondary antibody (1: 200, SA012, Auragene) was applied and incubated at 37°C for 1 hour in the dark. Nucleus was stained with DAPI in the dark for 5 min. After washing in PBS for 4 times, the image was visualized under a fluorescence microscope (Nikon, Tokyo, Japan).

### 2.10. TEM

Transmission electron microscopy was implemented to verify the form of cell death in FLS cells. Washing with PBS for 3 times, cultured cells were collected with trypsin and centrifuged at 1000 rpm for 5 min. Cell pellets were collected and prepared with 2.5% glutaraldehyde. The morphological change of cells was observed under TEM.

### 2.11. Hoechst and PI Staining

Cell pyroptosis was measured by Hoechst and PI staining. Briefly, the cells were cultured in 6-well plates for 24 h and challenged with LPS+ATP or saline. Staining with Hoechst 33342 and PI under the manufacturer's instruction (Solarbio, China), the cells with perforated membrane (PI permeable) were analyzed by a Zeiss microscope and ImageJ program.

### 2.12. LDH Release Assay

According to the manufacturer's protocols, a Lactate Dehydrogenase Assay Kit (Abcam, Cambridge, UK) was used to measure the release of LDH in supernatants following stimulation of cells.

### 2.13. ELISA

The level of IL-1*β* and IL-18 in supernatants of cultured cells following stimulation was detected using an ELISA kit (4A BIOTECH, China) under the manufacturer's instructions. The absorbance was measured at 450 nm.

### 2.14. Flow Cytometry

Annexin V-kFluor488/PI Detection Kit (KeyGen Biotech) was used to double stain cells according to the instructions. Quantification was then performed by flow cytometry.

### 2.15. Western Blot

The protein from tissue or cultured cell was extracted using RIPA buffer (R0010, Solarbio, China). Lysates were obtained by centrifugation at 4°C with 12000 rpm for 15 min. Protein levels were quantified with a BCA protein assay kit (Beyotime Biotechnology, China). Then, total protein was separated via SDS-PAGE and transferred to polyvinylidene fluoride (PVDF) membranes (Lot#K5NA8025F, Millipore, USA). Block membranes with 5% skim milk TBS-T for 1.5 h. Incubate them overnight at 4°C with primary antibodies against NLRP3 (1 : 1000, ET1610-93, Huabio), ASC (1 : 1000, DF6304, Affinity), caspase-1 (1 : 1000, ET1608-69, Huabio), IL-1*β* (1 : 1000, AF5103, Affinity), GSDMD (1 : 1000, AF4012, Affinity), and GAPDH (1 : 1000, 181602, Abcam). The membranes were then washed with TBS-T, followed by incubation with secondary antibodies at room temperature for 1.5 h. After washing 3 times with TBS-T, blot signals were visualized by chemiluminescent kit (Millipore, USA).

### 2.16. Real-Time PCR

According to the manufacturer's protocol, total RNA was isolated from cultured cells or synovial tissue using TRIzol (Invitrogen). Reverse transcription was synthesized via a first strand cDNA synthesis kit (Servicebio). qPCR was performed using a 2xSYBR Green qPCR Master Mix (Servicebio) and operated on ABI Prism 7500 system (Applied Biosystems, Life Technologies). The sequences of primers are listed in Table 1. The expression level of target genes was calculated by a 2^–*ΔΔ*Ct^ method after being normalized to GAPDH expression.

### 2.17. Statistical Analysis

Data are presented as the mean ± SD. For statistical analysis, independent samples *t*-test and one-way ANOVA followed by Bonferroni's post hoc test were used by SPSS and GraphPad Prism 7.

## 3. Results

### 3.1. Intra-Articular Injection of CFA Led to Synovitis of TMJ

Under H&E staining, synovial tissue showed several characteristic changes in the OA group compared to the sham group (Figure 1(b)), including significant thickening of synovial lining layer. Proliferated synovial lining presented typical villous hyperplasia into the cavity. Intensive infiltration of mononuclear cells, proliferation of dilated blood vessels, and scattered lipid droplets were also common. There were marked inflammatory and degenerative changes in the condylar cartilage. Collagen fibers on the surface of the condylar cartilage were edematous and loosened. In some samples, clefts were even formed in the fibrous or cartilage layer. The zone of proliferative layer, maturation layer, and hypertrophic layer was thinned in the OA group. However, erosion of subchondral bone was not observed. All features above proved that intra-articular injection of CFA can successfully induce TMJOA, especially synovial inflammation. These changes were not observed in the sham group, which is consistent with the other published researches [18].

### 3.2. Increased Expression of NLRP3 Inflammasome in the Synovium of TMJOA in Rats

**Figure 2:**
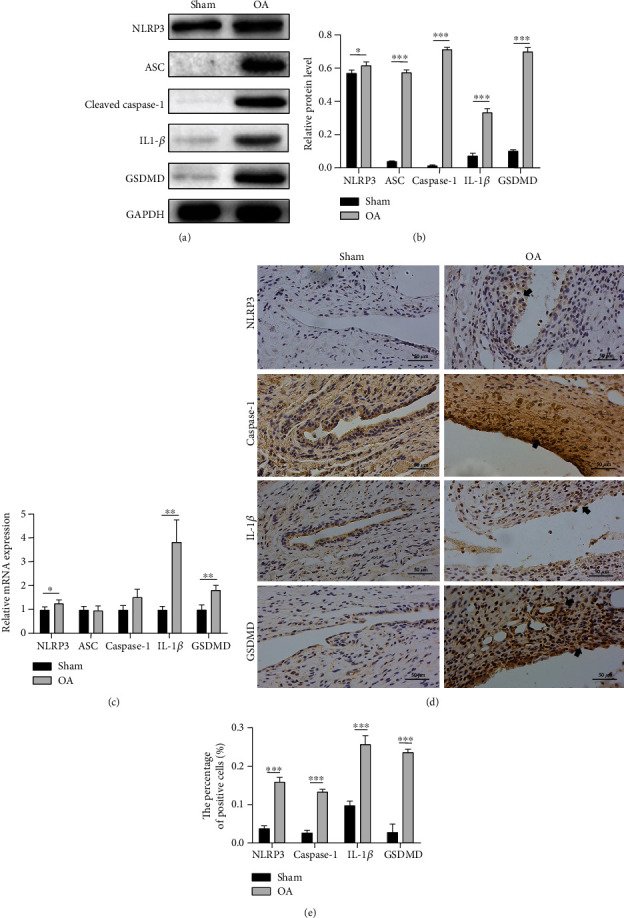
Elevated expression of molecules in TMJ synovium. (a, b) Western blotting results of NLRP3, ASC, cleaved caspase-1, IL-1*β*, and GSDMD in the sham and OA synovitis rats. (c) mRNA levels of NLRP3, ASC, caspase-1, IL-1*β*, and GSDMD expressed in TMJ synovium between the sham and OA rats. (d) Immunohistochemistry of TMJ slices from the sham or OA rats using anti-NLRP3, anti-caspase-1, anti-GSDMD, and anti-IL-1*β* antibodies, scale bar = 50 *μ*m. Positive cells are mainly around synovial membrane and vessels (black arrow). (e) Percentage of positive cells. Data in this figure are analyzed by independent samples *t*-test and presented as mean ± SD. ^∗^*P* < 0.05, ^∗∗^*P* < 0.01, and ^∗∗∗^*P* < 0.001.

Assembling of NLRP3 and caspase-1 by ASC is thought to be the key process activating inflammasome. Compared to the sham group, the protein and mRNA expressions of NLRP3 were elevated in the TMJOA synovial tissues (Figures 2(a)–2(c)). The protein level of cleaved capase-1 was significantly upregulated (Figures 2(a) and 2(b)). Inconsistently, the change of caspase-1 was not that obvious on the mRNA level (Figure 2(c)). The difference might result from the autocatalysis of pro-caspase-1 to form the active cleaved caspase-1. Inflammatory cytokine IL-1*β* and pyroptosis-specific protein GSDMD were also markedly increased in the OA rats, which were barely expressed in the sham group (Figures 2(a)–2(c)).

An increasing number of pyroptotic cells which express GSDMD were observed in the OA group by immunohistochemical staining, especially around synovial membrane and vessels (Figures 2(d) and 2(e)). The production of IL-1*β* NLRP3 and caspase-1 was also upregulated (Figures 2(d) and 2(e)). The result demonstrated that NLRP3 inflammasome-mediated pyroptosis was positively correlated with TMJOA synovitis in vivo.

### 3.3. Application of Inhibitors Ac-YVAD-cmk and MCC950 Ameliorated Pyroptosis and Synovial Inflammation of TMJOA

**Figure 3:**
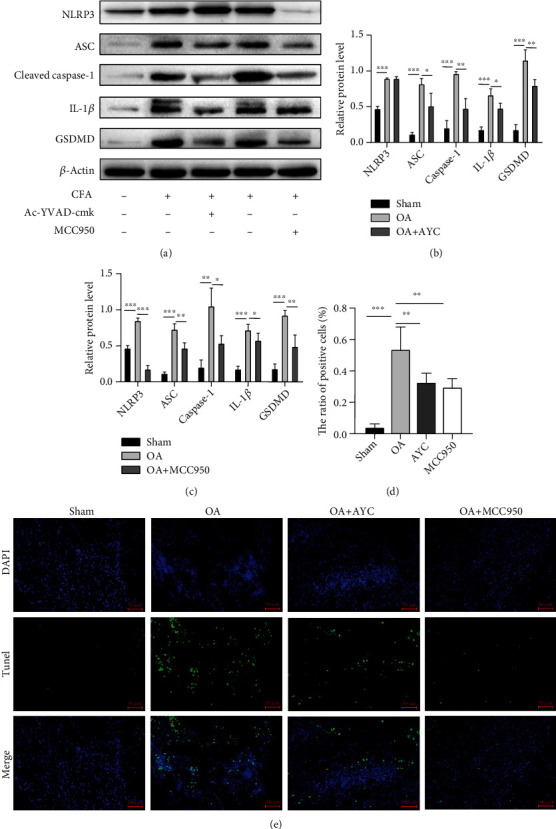
The biological effect of AYC and MCC950 on inflammasome- and pyroptosis-related molecules. (a, b) Western blotting results showing the protein levels of NLRP3, ASC, cleaved caspase-1, IL-1*β*, and GSDMD among the sham, OA, and AYC groups. (a, c) Levels of the above proteins among the sham, OA, and MCC950 groups. (d, e) TUNEL assay indicated that positive nuclei were increased markedly in the OA synovial tissue compared with the sham-treated group. Positive cells were reduced in the Ac-YVAD-cmk and MCC950 groups. Data in this figure are presented as mean ± SD and analyzed by parametric test. Statistical significance is determined with one-way ANOVA followed by Bonferroni's post hoc test. ^∗^*P* < 0.05, ^∗∗^*P* < 0.01, and ^∗∗∗^*P* < 0.001.

To further confirm the executor of pyroptosis, inhibitor Ac-YVAD-cmk (AYC) targeting caspase-1 was injected intra-articularly. We found that the protein level of IL-1*β*, GSDMD, caspase-1, and ASC was downregulated after AYC treatment. However, the change of NLRP3 was not observed (Figures 3(a) and 3(b)). The result proved that by interfering the cleavage of caspase-1, downstream maturation of IL-1*β* and GSDMD was restrained.

In addition, we used a highly selective inhibitor MCC950 targeting NLRP3 every 2 days. The results showed a significant suppression of NLRP3, caspase-1, IL-1*β*, and GSDMD expressions on TMJ synovium (Figures 3(a) and 3(c)). Both Ac-YVAD-cmk and MCC950 treatments can attenuate synovial inflammation and pyroptosis in TMJOA rats.

To further verify the function of inhibitors, TMJ tissues were sectioned and prepared for TUNEL assays. TUNEL assays revealed that the positive cells were significantly increased in the TMJOA region. Consistently, fewer positive cells were observed in the OA+AYC and OA+MCC950 groups when compared with the OA group (Figures 3(d) and 3(e)). The results above demonstrated that Ac-YVAD-cmk and MCC950 may have therapeutic effect in TMJ synovial inflammation via interfering with the activation process of NLRP3 inflammasome and inhibiting synoviocyte pyroptosis.

### 3.4. LPS+ATP-Induced Pyroptosis of THP-1 Cells

To further explore the possible interaction among TMJ synoviocytes, we then evaluated the effect of LPS+ATP on cultured THP-1 cells and rat FLS cells in vitro.

**Figure 4:**
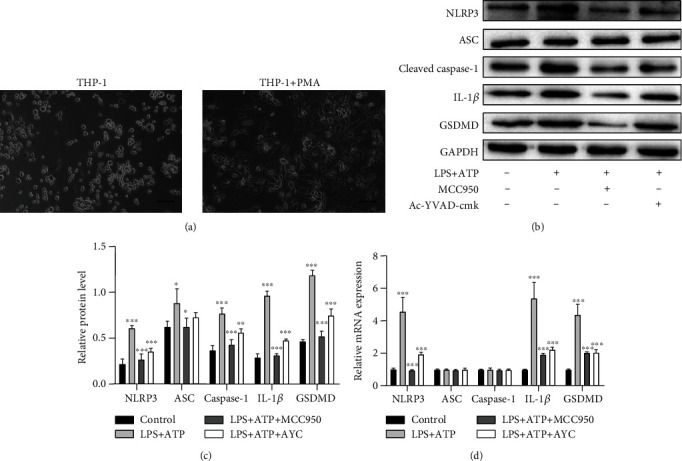
LPS+ATP-treated macrophages. (a) Altered morphology of THP-1 cells under PMA stimulation. Scale bar = 50 *μ*m. (b, c) Western blotting results of NLRP3, ASC, cleaved caspase-1, IL-1*β*, and GSDMD changes on protein levels in LPS-treated macrophages. (d) The results of qRT-PCR showed changes on mRNA levels among the control, LPS+ATP, LPS+ATP+MCC950, and LPS+ATP+AYC groups. Data are presented as mean ± SD and analyzed with one-way ANOVA followed by Bonferroni's post hoc test. ^∗^*P* < 0.05, ^∗∗^*P* < 0.01, and ^∗∗∗^*P* < 0.001.

The THP-1 cells are round suspension cells. Followed by stimulation with PMA for 48 h, activated macrophages were featured adherent and morphologically polygonal and long pseudopods (Figure 4(a)). Western blot and qRT-PCR analysis revealed that NLRP3, ASC, caspase-1, GSDMD, and IL-1*β* were upregulated in macrophage cell lysates treated by LPS+ATP compared to the control macrophages (Figures 4(b)–4(d)).

Inhibitory effects of Ac-YVAD-cmk and MCC950 in regulation of NLRP3 inflammasome activation were also explored on THP-1 macrophages. Pretreated with Ac-YVAD-cmk and MCC950 before ATP, notable inhibitory effect on inflammasome and GSDMD were observed in the above tests (Figures 4(b)–4(d)). This result was consistent with the other reported researches [8, 21–23].

### 3.5. The Activation of NLRP3 Inflammasome in the Coculture System

**Figure 5:**
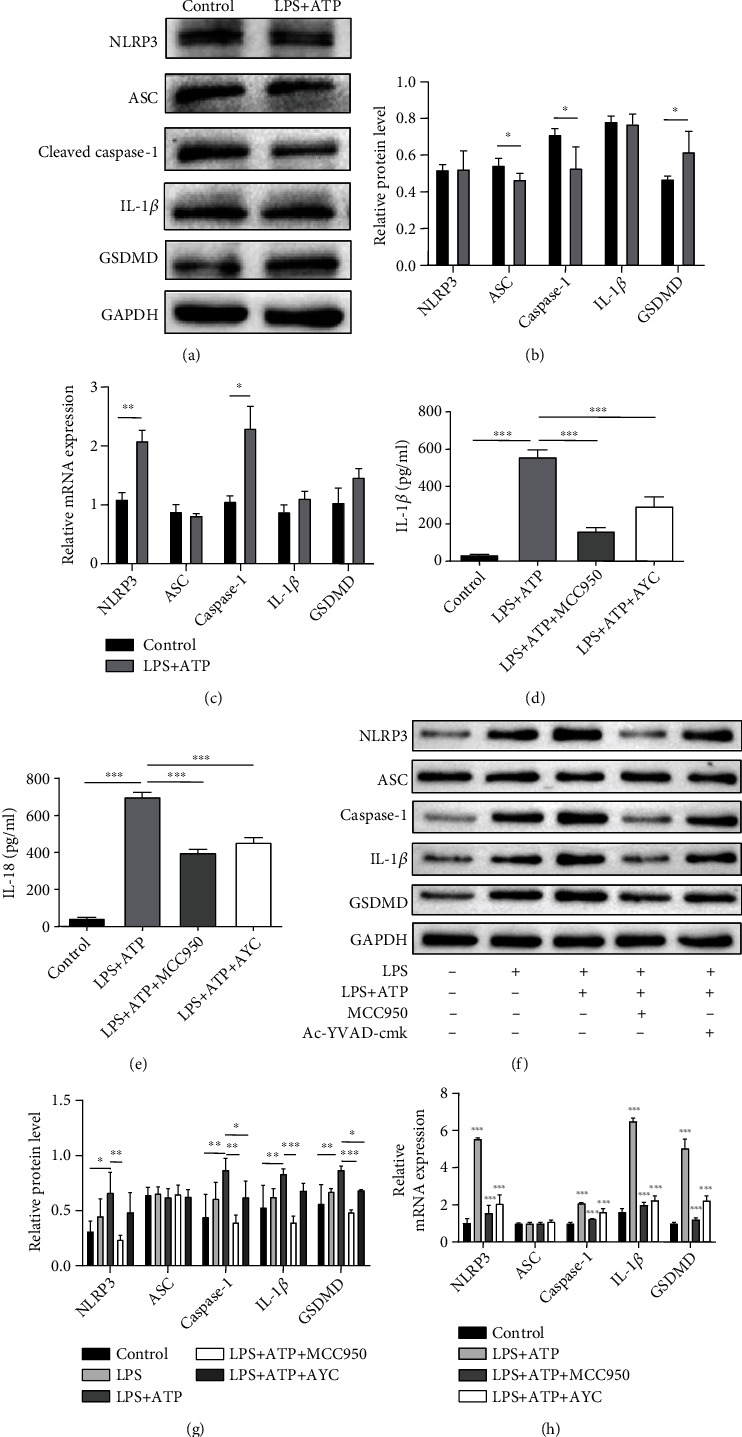
LPS+ATP stimulated NLRP3 inflammasome- and pyroptosis-related molecules in the cultured FLS cells alone and cocultured system. (a, b) When FLS cells were cultured alone, lysate was collected for western blot after being treated with LPS+ATP. (c) Total RNAs of FLS cells were extracted for detecting mRNAs of NLRP3, ASC, caspase-1, GSDMD, and IL-1*β*. Statistical analysis is run by independent samples *t*-test. (d, e) Secretion of IL-1*β* and IL-18 in the supernate increased when FLS cell was cocultured with active THP-1 cell. (f–h) Western blotting and qRT-PCR results of cell lysate indicated the growing expression of NLRP3, ASC, caspase-1, GSDMD, and IL-1*β* on protein and mRNA levels. Both the upregulation trend of the above tests after LPS+ATP intervention could be inhibited by MCC950 and AYC in the coculture system. Data are presented as mean ± SD; one-way ANOVA followed by Bonferroni's post hoc test was adopted for analysis. ^∗^*P* < 0.05, ^∗∗^*P* < 0.01, and ^∗∗∗^*P* < 0.001.

Fibroblast-like synovial cells are the major kind of synoviocytes in TMJ synovial tissue. We extracted and cultured primary TMJ-FLS cells from SD rats, and the third generation was used for our study. The classic combination of LPS+ATP was performed to induce pyroptosis. However, FLS did not react as we expected. No obvious activation of inflammasome was observed. Only slightly increased GSDMD level was observed in WB (Figures 5(a) and 5(b)). Interestingly, both mRNA levels of NLRP3 and caspase-1 increased, which were conflicting with the protein change (Figure 5(c)).

However, when FLS cells were cocultured with THP-1, secretion of IL-1*β* (Figure 5(d)) and IL-18 (Figure 5(e)) in the supernate, as well as inflammasome-related components in cell lysate (Figures 5(f) and 5(g)), was detected to be significantly increased. The qRT-PCR results are also consistent with the trend of protein (Figure 5(h)). The expression of protein and RNA levels can also be inhibited after application of MCC950 and Ac-YVAD-cmk to the culture medium to restrain the activity of inflammasomes in the FLS cells (Figures 5(f)–5(h)).

### 3.6. Pyroptosis Changes of FLS Cells in the Cocultured System

**Figure 6:**
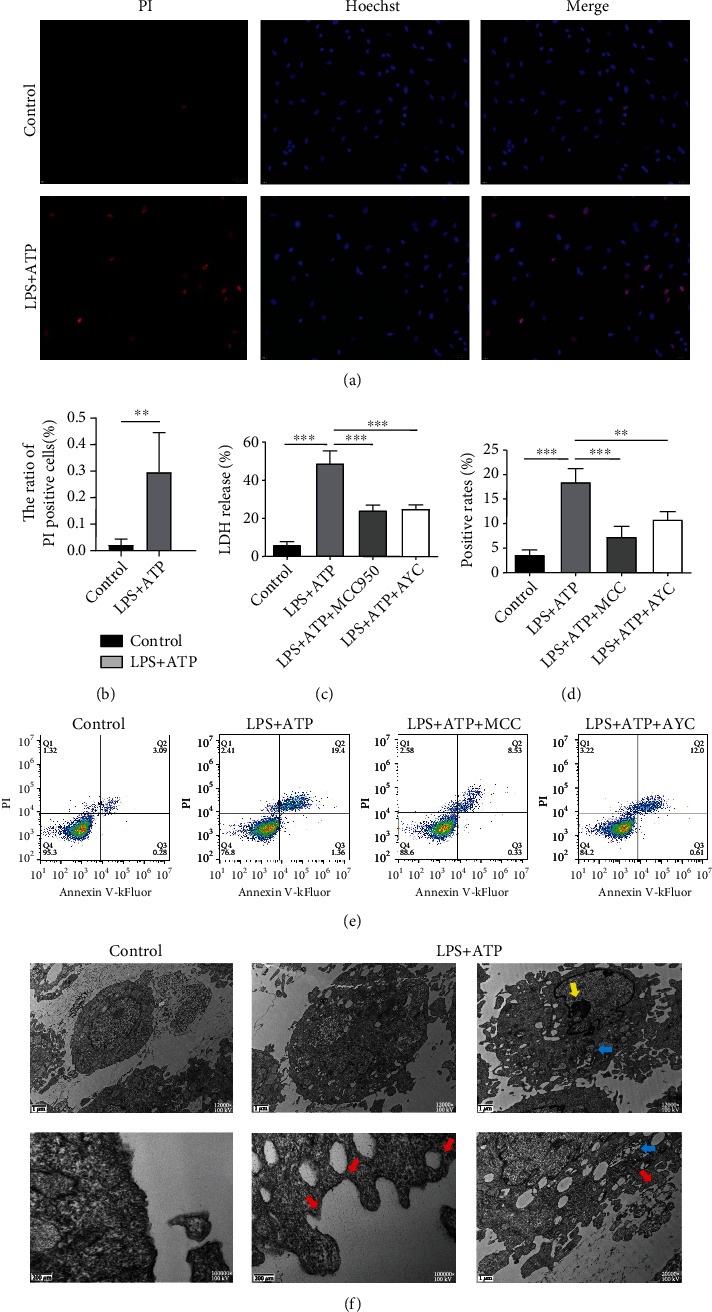
Detection of pyroptosis in fibroblast-like synovial cells of coculture system. Cocultured FLS cells were challenged with LPS for 12 h and ATP for 2 h. Cell exposed to saline was as the control group. The MCC950 group and Ac-YVAD-cmk group were simultaneously added inhibitors to FLS cells. (a) PI- and Hoechst 33342-stained FLS cells in the control and LPS+ATP groups. (b) Quantification of PI-positive cells. Independent samples *t*-test. (c) The release of LDH was measured among the control, LPS+ATP, LPS+ATP+MCC950, and LPS+ATP+AYC groups. (d, e) Cells double stained by PI and kFluor488 for flow cytometry. One-way ANOVA followed by Bonferroni's post hoc test was used. ^∗^*P* < 0.05, ^∗∗^*P* < 0.01, and ^∗∗∗^*P* < 0.001. (f) Representative transmission electron micrographs of FLS cells. Compared with control group, FLS cells exhibited pyroptotic features: extensive membrane bubbles and pores (red arrow); chromatin margination and condensation (yellow arrow); and swollen mitochondria with collapsed cristae (blue arrow).

Pyroptotic FLS cells were double stained by propidium iodide (PI)/Hoechst 33342 for further study. PI-positive cells were upregulated compared with the control group (Figures 6(a) and 6(b)). Lactate dehydrogenase (LDH) activity in cell supernate was examined. The amount of LDH release markedly increased after LPS+ATP intervention (Figure 6(c)), which indicated the loss of membrane integrity during pyroptosis. Flow cytometric analysis by double-labeled annexin V-kFluor488 and PI detected an increasing number of PI-positive FLS cells (Figures 6(d) and 6(e)) and few apoptotic-like (annexin-V+/PI−) cells. Given that pyroptosis was inflammasome-mediated and caspase-1-dependent cell death, we added inhibitors MCC950 and Ac-YVAD-cmk. The percentage of PI-positive cells was significantly reduced (Figures 6(d) and 6(e)). Moreover, ultrastructural analysis by transmission electron microscopy (TEM) also showed the variation of FLS morphology including chromatin condensation, swollen mitochondria, dispersed trans-Golgi network (dTGN), bubbles, and pore formation of plasma membrane following stimulation (Figure 6(f)).

The result suggested that THP-1-differentiated macrophages may activate fibroblast-like synoviocytes in inflammatory process and amplify the inflammation response. Soluble factors released by the macrophage appear to be, at least in part, responsible for the fibroblast activation.

## 4. Discussion

Our study demonstrated that NLRP3 inflammasome-mediated pyroptosis was involved in rat TMJOA synovitis. Moreover, we found that activated NLRP3 inflammasome and consequent GSDMD pore formation were augmented in FLS cell and macrophage cocultured system in vitro. Inhibiting NLRP3 and caspase-1 by MCC950 and Ac-YVAD-cmk could suppress pyroptosis and subsequent inflammatory response in the TMJOA rats and cultured synoviocytes. Our study explored the possible role of NLRP3 inflammasome-mediated pyroptosis in the development of TMJOA.

TMJOA is one of the most common TMJ disorders in the world. Increasing evidence points out that synovitis occurs at initial stage of TMJOA and plays a critical role in the entire process, which suggest that targeting the synovial inflammation could be a promising strategy treating OA. In this study, we used a commonly used CFA intra-articular injection method to establish a reproducible TMJOA rat model [18–20, 24]. Typical pathological changes of synovitis were reproduced with proliferated synovial lining and infiltrated inflammatory cells.

TMJOA is an inflammatory disease featured with synovial proliferation, immune cell infiltration, and numerous proinflammatory cytokine release, including IL-1*β*, IL-6, IL-18, TNF-*α*, cyclooxygenase-2 (COX-2), matrix metalloproteinases, and prostaglandin E2 (PGE2) [25–27]. It is widely acknowledged that IL-1*β* is a crucial inflammatory mediator corresponding to osteoarthritis severity. IL-1*β* could not only initiate synovitis during TMJOA but also accelerate the differentiation of osteoclasts, consequently inducing cartilage degradation [28]. Nevertheless, the origin of IL-1*β* and how it rapidly escalates the inflammatory response in TMJOA have not been thoroughly illustrated.

Recently, pyroptosis, a modality of programmed cell death, was observed in multiple diseases. Canonical pyroptosis relies on the cleaved caspase-1, leading to substrate gasdermin D cleavage, cell lysis, and release of IL-1*β* [23, 25, 29], which, in inflammatory diseases, could cause a cascade of inflammatory reaction via further activation of resident cells and recruiting more inflammatory cells to infiltrate [12]. NLRP3 inflammasome-mediated synoviocyte pyroptosis was reported to be involved in the progress of knee osteoarthritis [16, 30–32]. Our preliminary experiment proved that the NLR family including NLRP3 complex was detected by mRNA sequencing in rat TMJ synovial tissue.

We proposed that the NLRP3-mediated pyroptosis is involved in the TMJOA synovitis and may aggravate the subsequent inflammation. Consistently, our study showed that NLRP3 acting as a proinflammatory mediator was found to be expressed in rat TMJOA synovial tissue, along with the elevated activity of caspase-1, IL-1*β*, and GSDMD [25]. Also, an increasing number of pyroptotic cells were seen in rat synovitis tissue. Besides, aiming NLRP3 and caspase-1 inhibitors suppressed IL-1*β* and GSDMD expressions, as well as the existence of pyroptotic cells. Therefore, we proved that the NLRP3 inflammasome-mediated pyroptosis is involved in the process of TMJOA synovitis.

In vitro, the combination of LPS+ATP was a widely accepted method to induce cell pyroptosis. Synovial fibroblasts and macrophages are major cell populations of synoviocytes [33]. The effect on macrophage was replicated in our study, which was coincident with the other reported researches [8, 10, 23]. However, the stimulation of FLS cells was rarely reported. Our study revealed that FLS alone hardly exerted inflammasome activation. It could only mediate obvious inflammation when cocultured with macrophages. The activated macrophage might provide crucial soluble factors to accelerate assembling of the NLRP3 inflammasome in FLS cells. Activated fibroblasts may contribute to synovial proliferation, amplified inflammation, osteoclast differentiation, and cartilage degradation when cocultured with macrophages [34]. It has been reported that cocultured TMJ fibroblast-like cells and macrophages without cell-cell contact augment monocyte chemoattractive protein-1 (MCP-1) production and expanded inflammation [4]. Infiltrated immune cells including macrophages activate an inflammatory profile in fibroblasts via nuclear factor-*κ*B (NF-*κ*B). Fibroblasts recruit more immune cells by producing inflammatory factors, propagating NF-*κ*B signaling, and leading to a positive feedback loop [35]. The underlying interaction between FLS and MLS is still under investigation.

Inflammatory stimulus triggered NLRP3 inflammasome assembly, turning pro-caspase-1 into cleaved caspase-1, and eventually induced pyroptosis occurrence. In this study, the application of NLRP3 inhibitor MCC950 blocked NLRP3 inflammasome activation both in vivo and in vitro. The levels of cleaved caspase-1, IL-1*β*, and GSDMD in the synovial tissue were decreased. TMJOA synovitis improved with ameliorated synovial hyperplasia and less inflammatory cell infiltration. Application of a selective inhibitor of caspase-1, Ac-YVAD-cmk, could also restrain inflammatory response by suppressing the maturation of IL-1*β* and cell pyroptosis. However, NLRP3 levels in the synovial tissue are almost unchanged. It indicted that NLRP3 might activate immediately after stimulus, but the occurrence of pyroptosis still requires cleavage of caspase-1. Overall, both Ac-YVAD-cmk and MCC950 have exerted protective effect on synovitis of TMJ. In comparison, delivery of MCC950 revealed a more orderly arranged synovial lining and less infiltrated cells.

Extensive cell pyroptosis with pore formation was detected in our study. N-Terminal fragment of GSDMD is known capable of drilling on the plasma membrane [14, 25, 36]. In contrast to other forms of programmed cell death, pyroptosis is more of a combination of apoptosis and necrosis [13]. Morphologically, pyroptosis involves pore formation in the plasma membrane. When intracellular stress overwhelms innate membrane-repair mechanisms, cells rupture and form surrounding vesicle consequently. The nucleus condenses but maintains the integrity. Pyroptosis promotes the rupture of plasma membrane, contributing to the release of proinflammatory mediators and inflammatory cytokines [23, 37]. A recent study discovered that dispersed trans-Golgi network (dTGN) existed as vesicles in the plasma, serving as a scaffold for NLRP3 to aggregate, which is essential for ASC interaction and downstream signaling cascade [38].

There are some limitations in our study. Firstly, although THP-1 cell was widely used in inflammasome study, it cannot fully mimic the effect of MLS in our coculture system. Rat-derived macrophages might be a better approach. Also, to have a better insight into the pathogenesis of TMJOA, the interaction between fibroblasts and macrophages needs to be further explored.

To sum up, our findings provide evidence that assembly of NLRP3 inflammasome participates in the development of TMJOA, especially in synovial inflammation, which induces synoviocyte pyroptosis and propagates inflammatory response. MLS may interact with FLS contributing to the inflammation cascade and trigger subsequent destruction. Intervention in NLRP3 inflammasome assembly using MCC950 and Ac-YVAD-cmk could suppress pyroptosis and inflammation, which may further lead to the prevention of tissue damage.

## 5. Conclusion

The study identifies that NLRP3-mediated pyroptosis is closely related to TMJOA synovitis. MCC950 and Ac-YVAD-cmk targeting NLRP3 inflammasome assembly could ameliorate inflammation in TMJ. Further investigations into this new mechanism in the pathogenesis of TMJOA could be a promising direction.

## Figures and Tables

**Table 1:** Sequences of primers.

Gene	Forward primer	Reverse primer
NLRP3	5′-CTAGCCACGCTAATGATCGACT-3′	5′-CCACTCCTCTTCAATGCTGTCT-3′
ASC	5′-TGGATGCTCTGTACGGGAAG-3′	5′-CCAGGCTGGTGTGAAACTGAA-3′
Caspase-1	5′-CAAGGTCCTGAAGGAGAAGAGAA-3′	5′-TTGTTCAGCACCCTTGTCTGTA-3′
IL-1*β*	5′-CGCCAGTGAAATGATGGCTTAT-3′	5′-TAGTGGTGGTCGGAGATTCGTA-3′
GSDMD	5′-CTGCTCCATGAGAGGCACCTG-3′	5′-GTGACTTCCACCTCCTTCTGTG-3′
GAPDH	5′-CTGGGCTACACTGAGCACC-3′	5′-AAGTGGTCGTTGAGGGCAATG-3′

## Data Availability

The data used to support the findings of this study are available from the corresponding author upon request.
